# Whole-genome sequences of 20 *Bacillus cereus* strains isolated from various environments

**DOI:** 10.1128/mra.01129-25

**Published:** 2026-01-29

**Authors:** Emilie Viktoria Skriver, Tessa Susanne Canoy, Eoghan Thomas Reilly, Elissavet Gkogka, Daniella Lucena, Susanne Knøchel, Henriette Lyng Røder

**Affiliations:** 1 Department of Food Science, University of Copenhagen 4321, Copenhagen, Denmark; 2 Arla Innovation Center, Arla Foods amba, Aarhus, Denmark; Fluxus Inc., Sunnyvale, California, USA

**Keywords:** *Bacillus cereus*, whole-genome sequencing, Oxford nanopore sequencing, food safety

## Abstract

We report whole-genome sequences of 20 *Bacillus cereus sensu lato* strains from plant-based fermentations, foodborne outbreaks, environmental sources, and dairy plants. Genomic DNA was extracted and sequenced using Oxford Nanopore Technology. These complete assemblies provide high-quality resources for taxonomic resolution and comparative analyses of pathogenicity within the *B. cereus* group.

## ANNOUNCEMENT

The *Bacillus cereus* group comprises genetically diverse bacteria, including strains capable of producing enterotoxins and cereulide, posing a risk to food safety (1, 2). Whole-genome sequencing was performed on 20 strains from various sources, with provenance and reference publications detailed in Table 1. These genomic resources will enable taxonomic resolution and comparative analyses of pathogenicity within the *B. cereus* group. Restreaked cryostock cultures (–80°C; brain heart infusion [BHI] broth [HiMedia, Germany] with 20% glycerol) were repropagated in BHI broth and incubated 48 h at 30°C at 200 rpm. Two-step centrifugation (3,900 × *g* for 10 min, decanting, addition of 1 mL 0.9% NaCl solution, and 13,800 × *g* for 3 min) enabled the collection of cells. Genomic DNA (gDNA) was extracted from bacterial pellets using the bead-beat micro AX Gravity kit following the instructions of the manufacturer (A&A Biotechnology, Poland; catalog number 106-100). The total gDNA was quantified using a Qubit fluorometer (Invitrogen; 1× double-stranded DNA high-sensitivity and broad-range assay kits; Thermo Fisher, USA).

Sequencing libraries were prepared without size selection using the Oxford Nanopore Technologies’ Native Barcoding Kit 96 V14, UK (SQK-NBD114.96) and rapid barcoding protocol NBE_9171_v114_revO_15sep2022, following the manufacturer’s instructions. Sequencing was performed on PromethION 2 Solo (PRO-SEQ002) for 72 h using standard settings. Reads were base-called (Dorado v7.1.4), filtered (Chopper v0.9.1; --quality 15 --minlength 1,000) (3), and assembled with Hybracter (v0.11.2; –no_medaka –skip_qc) (4). Briefly, contigs were assembled using Flye (v2.9.2) at 100× coverage and 4.8 Mb minimum chromosome size (5). Plasmids were assembled with Plassembler (v1.6.2) (6), circularized chromosomes reoriented to dnaA using Dnaapler (v1.1.0) (7), and annotated with PGAP (v6.10) (8). Taxonomy was tentatively assessed through comparative analyses with reference type strains, using the average nucleotide identity based on MUMmer (ANIm) scores (JSpeciesWS v3.9.8; 96% threshold) (9), and by Digital DNA-DNA hybridization (dDDH) values (TYGS; formula d4; 70% species delineation threshold) (10, 11) (Table 1). Standard settings were used for all software except where otherwise noted.

**TABLE 1:** Accession information, characteristics, assembly statistics, and genomic features of the 20 *B. cereus* genomes

Strain designation	Origin	Taxonomy (reference strain)	dDDH (%)	ANIm ON-seq (%)	GenBank accession no.	SRA accession no.	Assembly size (bp)	No. of raw reads	G+C content (%)	Coverage (X)	No. of contigs	ONT N50	No. of predicted genes (PGAP)	Completeness	Reference
F6H3	Locust bean fermentation, Benin, Africa	*Bacillus cereus^a^*^,*t*^ (ATCC 14579)	45.8	92.09 ^*b*^	GCA_052947535.1	SRX30618984	5,563,324	34,103	35.5	23.93	6	11,431	5,770	Complete	(12)
Y48H10	Locust bean fermentation, Benin, Africa	*Bacillus cereus^c^*^,*t*^ (ATCC 14579)	45.8	92.09^*b*^	GCA_052947495.1	SRX30618985	5,548,987	38,561	35.5	44.33	4	13,293	5,751	Complete	(12)
Ba18H2	Locust bean fermentation, Benin, Africa	*Bacillus cereus^d^*^,*t*^ (ATCC 14579)	45.8	92.09^*b*^	GCA_052947735.1	SRX30618986	5,559,855	42,079	35.5	44.05	5	11,813	5,761	Complete	(12)
SDA GR177	Raw milk, dairy plant, Sweden	*Bacillus cereus^e^*^,*t*^ (ATCC 14579)	45.7	92.09^*b*^	GCA_052948155.1	SRX30618987	5,535,842	181,101	35.5	84.79	2	12,976	5,735	Complete	(13)
SDA MA60	Raw milk, dairy plant, Sweden	*Bacillus cereus^f^* (ATCC 14579)	45.7	92.06^*b*^	GCA_052947565.1	SRX30618988	5,641,419	247,071	35.5	85.55	4	12,581	5,872	Complete	(13)
NVH 0075-95	Food-borne outbreak, Norway	*Bacillus cereus^g^* (ATCC 14579)	45.7	92.08^*b*^	GCA_052947775.1	SRX30618995	6,044,751	1,101,359	35.3	78.99	4	10,152	6,287	Complete	(14)
F4810/72	Cooked rice, food borne outbreak, UK	*Bacillus cereus^h^*^,*t*^ (ATCC 14579)	45.8	92.09^*b*^	GCA_052947835.1	SRX30618998	5,584,430	193,461	35.5	86.02	3	12,196	5,818	Complete	(15)
F4430/73	Foodborne outbreak, UK	*Bacillus cereus^i^* (ATCC 14579)	45.7	92.06^*b*^	GCA_052947745.1	SRX30618990	5,637,527	173,733	35.5	85.01	4	11,389	5,866	Complete	(16)
F3351/87	Foodborne outbreak, UK	*Bacillus cereus^j^*^,*t*^ (ATCC 14579)	45.7	92.06^*b*^	GCA_052947675.1	SRX30619000	5,638,718	45,117	35.5	48.82	4	12,994	5,873	Complete	(17)
NC7401	Foodborne outbreak, Japan	*Bacillus cereus^k^*^,*t*^ (ATCC 14579)	45.7	92.08^*b*^	GCA_052947595.1	SRX30618997	5,509,155	445,663	35.5	84.38	4	4341	5,735	Complete	(18)
LWL3	Foodborne outbreak, unknown region/country	*Bacillus cereus^l^* (ATCC 14579)	44.2	91.68^*b*^	GCA_052947655.1	SRX30618993	5,686,534	452,576	35.3	84.01	2	15,894	5,841	Complete	(19)
ISI2489	Rice protein, unknown region/country	*Bacillus cereus^m^* (ATCC 14579)	45.7	92.08^*b*^	GCA_052947815.1	SRX30618991	5,549,695	495,903	35.5	85.65	3	7,505	5,726	Complete	Arla culture collection
NS117	Norway spruce tree, Ruotsinkylä, Finland	*Bacillus cereus^n^*^,*t*^ (ATCC 14579)	45.6	92.04^*b*^	GCA_052947555.1	SRX30618999	5,746,703	95,046	35.4	82.36	6	11,399	5,959	Complete	(20)
LKT1/1	Filler material of a building, Finland	*Bacillus cereus^o^*^,*t*^ (ATCC 14579)	45.6	92.06^*b*^	GCA_052947855.1	SRX30618983	5,690,232	46,673	35.5	22.09	3	11,921	5,906	Complete	(21)
F4433/73	Meatloaf, Berkeley, California, USA	*Bacillus cereus^p^* (ATCC 14579)	46.5	91.68^*b*^	GCA_052947715.1	SRX30618981	5,433,211	560,610	35.5	88.57	2	11,499	5,616	Complete	(22)
O-216	Milk, Danish Dairy, Denmark	*Bacillus cereus^q^* (ATCC 14579)	44.8	91.89^*b*^	GCA_052947615.1	SRX30618982	5,580,549	243,767	35.4	84.56	3	12,677	5,829	Complete	(23)
59	Cream, Danish Dairy, Denmark	*Bacillus cereus^r^* (ATCC 14579)	44.2	91.72^*b*^	GCA_052947635.1	SRX30618994	5,532,701	201,062	35.5	78.25	6	7477	5,767	Complete	(24)
A536	Raw milk, silo tank, Sweden	*Bacillus cereus^s^* (ATCC 14579)	38.3	89.96^*b*^	GCA_052947695.1	SRX30618989	6,122,672	790,395	35.3	78.65	4	7,348	6,409	Complete	(13)
MC67	Sandy soil, Møn, Denmark	*Bacillus mycoides^t^* (DSM 2048)	58.8^*b*^	95^*b*^	GCA_052947795.1	SRX30618996	5,782,341	165,096	35.5	82.38	4	12,149	5,978	Complete	(25)
MC118	Sandy soil, Møn, Denmark	*Bacillus mycoides^t^* (DSM 2048)	58.8^*b*^	95^*b*^	GCA_052947515.1	SRX30618992	5,745,487	397,907	35.5	82.45	3	11,244	5,933	Complete	(25)

^*a*^ Classified as *B. cereus*, but more closely related to *Bacillus paranthracis *(reference genome Mn5; Digital DNA-DNA hybridization [dDDH], 77.5 %, ANIm, 97.62%).

^*b*^ Value is below the species threshold (<70% dDDH or <96% ANIm).

^*c*^ Classified as *B. cereus*, but more closely related to *Bacillus paranthracis *(reference genome Mn5; dDDH, 77.6%, ANIm, 97.62%).

^*d*^ Classified as *B. cereus*, but more closely related to *Bacillus paranthracis *(reference genome Mn5; dDDH, 77.5%, ANIm, 97.62%).

^*e*^ Classified as *B. cereus*, but more closely related to *Bacillus paranthracis *(reference genome Mn5; dDDH, 77.5%, ANIm, 97.62%).

^*f*^ Classified as *B. cereus*, but more closely related to *Bacillus paranthracis *(reference genome Mn5; dDDH, 77.5%, ANIm, 97.62%).

^*g*^ Classified as *B. cereus*, but more closely related to *Bacillus paranthracis *(reference genome Mn5; dDDH, 77.4%, ANIm, 97.61 %).

^*h*^ Classified as *B. cereus*, but more closely related to *Bacillus paranthracis *(reference genome Mn5; dDDH, 77.4%, ANIm, 97.62%).

^*i*^ Classified as *B. cereus*, but more closely related to *Bacillus paranthracis *(reference genome Mn5; dDDH, 77.5%, ANIm, 97.62%).

^*j*^ Classified as *B. cereus*, but more closely related to *Bacillus paranthracis *(reference genome Mn5; dDDH, 77.4%, ANIm, 97.62%).

^*k*^ Classified as *B. cereus*, but more closely related to *Bacillus paranthracis *(reference genome Mn5; dDDH, 77.2%, ANIm, 97.61 %).

^*l*^ Classified as *B. cereus*, but more closely related to *Bacillus pretiosus *(reference genome SAICEU11T; dDDH, 73.3%, ANIm, 97.1%) and *Bacillus wiedmannii *(reference genome FSL W8-0169; dDDH 68.4%, ANIm 96.43%).

^*m*^ Classified as *B. cereus*, but more closely related to *Bacillus paranthracis *(reference genome Mn5; dDDH, 77.8 %, ANIm, 97.64 %).

^*n*^ Classified as *B. cereus*, but more closely related to *Bacillus paranthracis *(reference genome Mn5; dDDH, 77.3 %, ANIm, 97.61 %).

^*o*^ Classified as *B. cereus*, but more closely related to *Bacillus paranthracis *(reference genome Mn5; dDDH, 77.5%, ANIm, 97.62%).

^*p*^ Classified as *B. cereus*, but more closely related to *Bacillus tropicus *(reference genome N24; dDDH, 67.4%, ANIm, 96.26 %).

^*q*^ Classified as *B. cereus*, but more closely related to *Bacillus pacificus *(reference genome EB422; dDDH, 99.2 %, ANIm, 99.94 %).

^*r*^ Classified as *B. cereus*, but more closely related to* Bacillus pretiosus* (reference genome SAICEU11T; dDDH, 69.8%, ANIm, 96.7%) and *Bacillus wiedmannii *(reference genome FSL W8-0169; dDDH 69.3%, ANIm, 96.56%).

^*s*^ Classified as *B. cereus*, but more closely related to *Bacillus mycoides *(reference genome DSM 2048; dDDH, 94.8%, ANIm, 97.23%).

^*t*^ Previous reported to produce the emetic toxin cereulide.

Long-read sequencing technologies enabled the generation of 20 high-quality genome assemblies. Most isolates were previously classified as *B. cereus sensu stricto* (*n* = 18) or *B. mycoides* (*n* = 2). However, our dDDH and ANIm analysis (see Table 1) suggests tentative matches to six *B. cereus sensu lato* species (*B. paranthracis, B. tropicus*, *B. pacificus*, *B. pretiosus*, *B. wiedmannii,* and *B. mycoides*), reflecting ongoing revisions in *B. cereus* taxonomy (26). These complete long-read genomes provide a strong resource for future formal taxonomic assignment, comparative genomics, and understanding species-level diversity within the *B. cereus* group.

## Data Availability

The whole genomes announced here have been deposited in GenBank under the accession no. listed in Table 1.
